# Editorial Changes

**Published:** 1849-04

**Authors:** 


### THE WESTERN JOURNAL.

THIRD SERIES.—VOL. III.—NO. IV.

LOUISVILLE, APRIL 1, 1849.

### EDITORIAL CHANGES.

Our readers cannot have failed to remark that the names of our associates, Dr. Drake and Dr. Colescott, have not for several months past appeared on the cover of the Journal. Weary of the task, and having the prospect of more profitable employment for their leisure hours, these gentlemen determined to abandon it at the beginning of the present year. We parted with them reluctantly, but have no right to complain that they were not disposed to continue any longer their connection with a work where the labor is great and the rewards small. Dr. Theodore S. Bell, who was associated with the enterprise in its incipiency, returns to it in their room. As a spirited, vigorous writer, Dr. Bell is well known to the profession. His industry is untiring, and he returns to the editorial tripod again with the experience of more than eleven years.

